# Supplementary figures and images for: Bitcoin: A life in crises
Source: PLoS One. 2022 Sep 30;17(9):e0274165. doi: 10.1371/journal.pone.0274165 (PMC9524670; doi:10.1371/journal.pone.0274165)

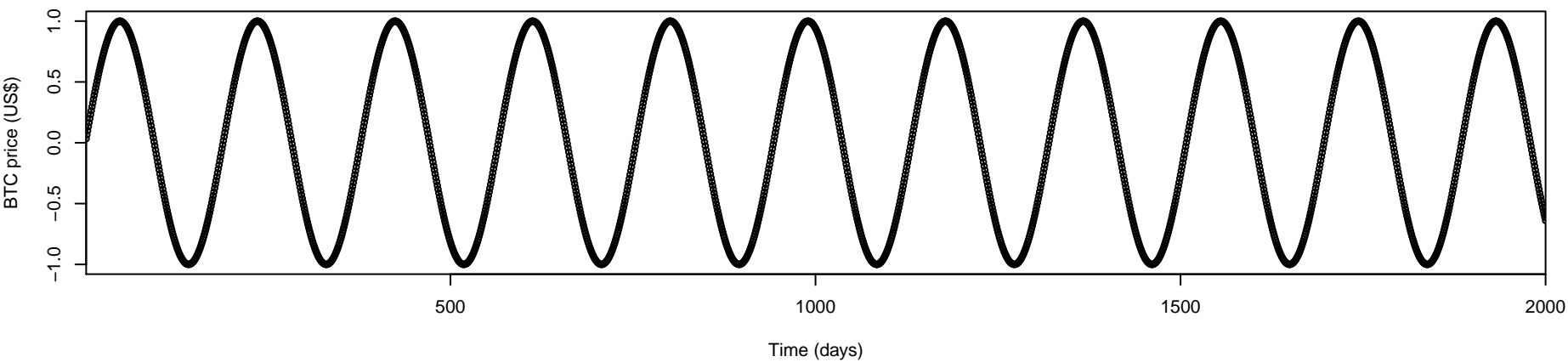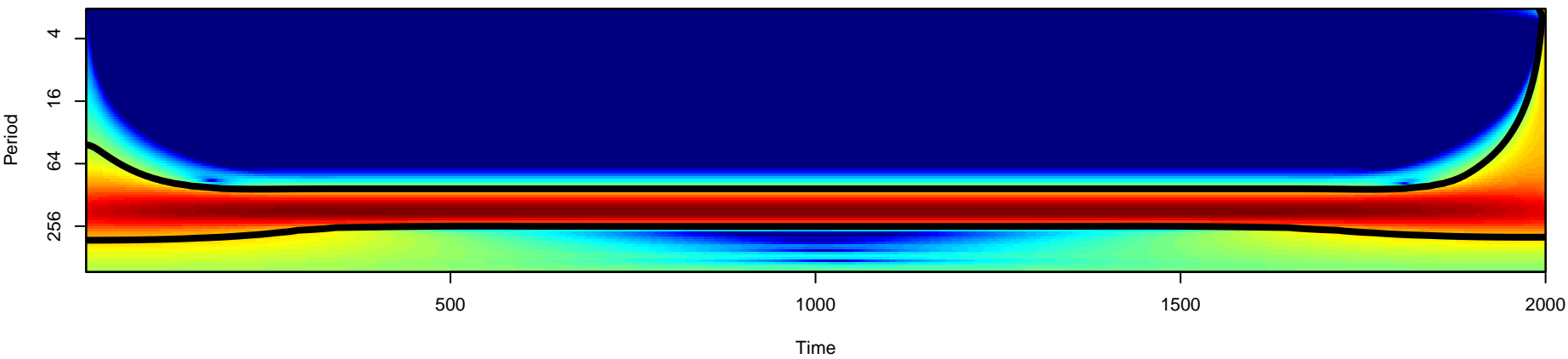

Supplement: S1 Fig — (PDF) [file pone.0274165.s001.pdf]

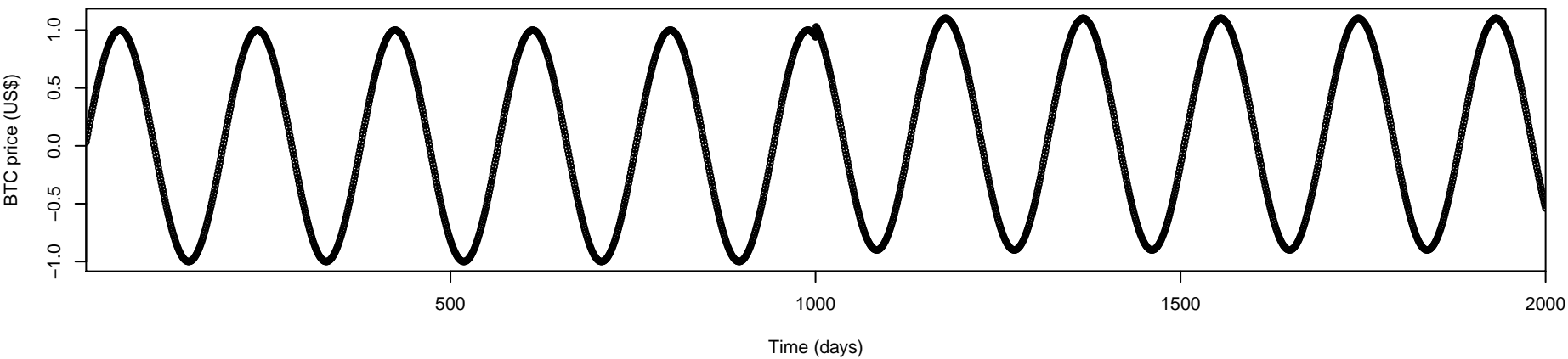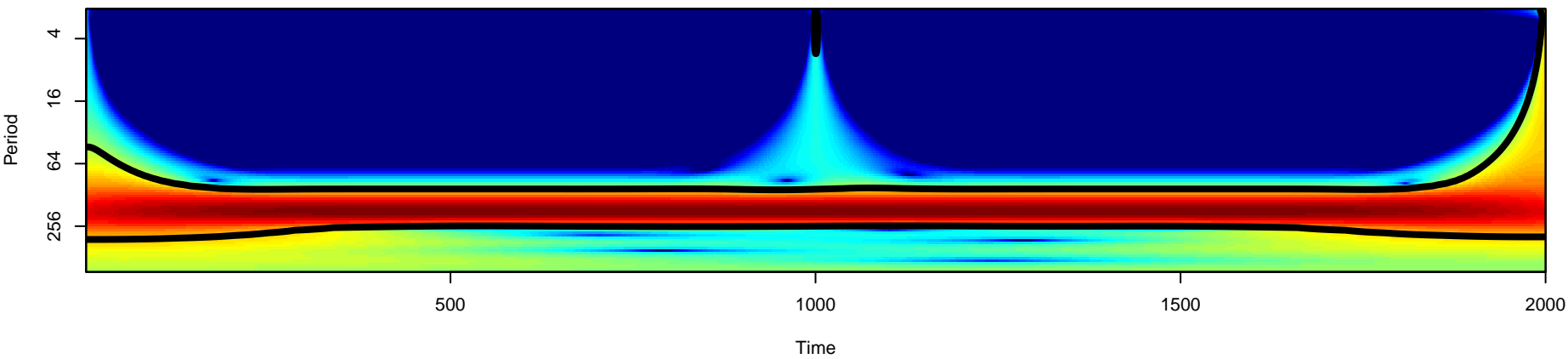

Supplement: S2 Fig — (PDF) [file pone.0274165.s002.pdf]

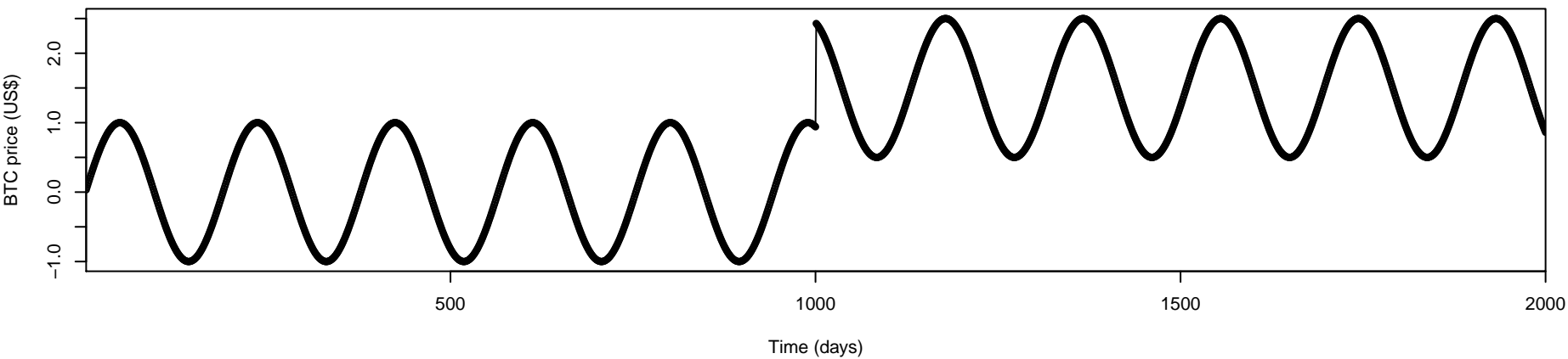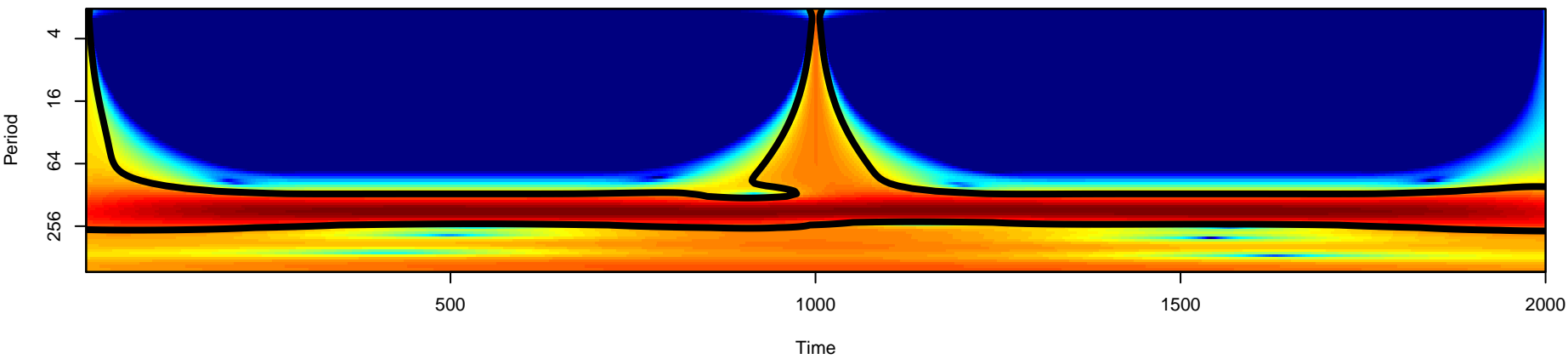

Supplement: S3 Fig — (PDF) [file pone.0274165.s003.pdf]

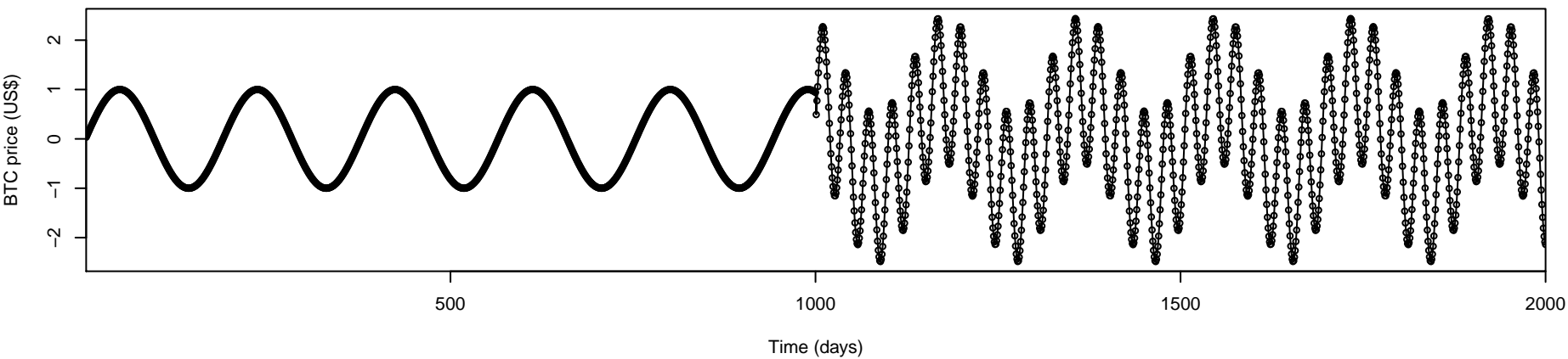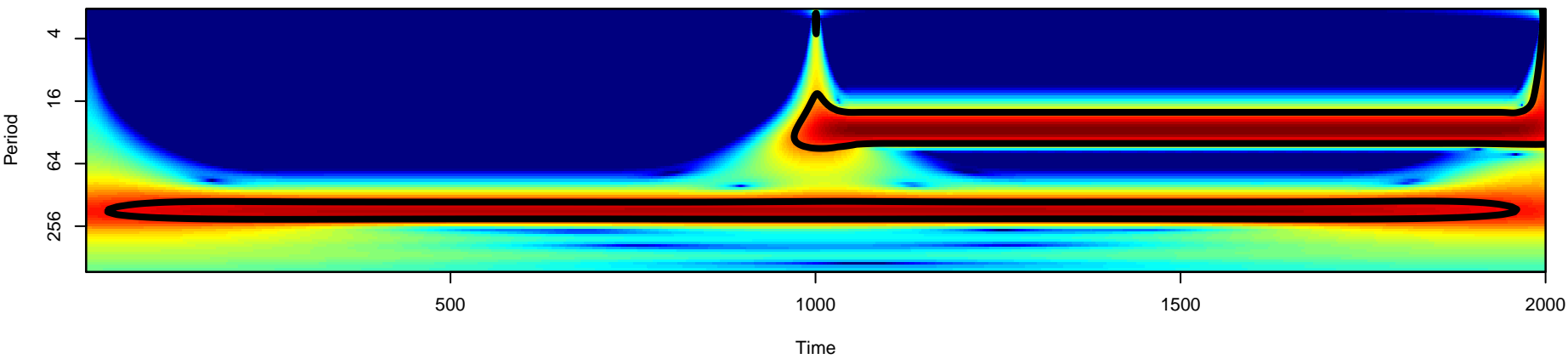

Supplement: S4 Fig — (PDF) [file pone.0274165.s004.pdf]

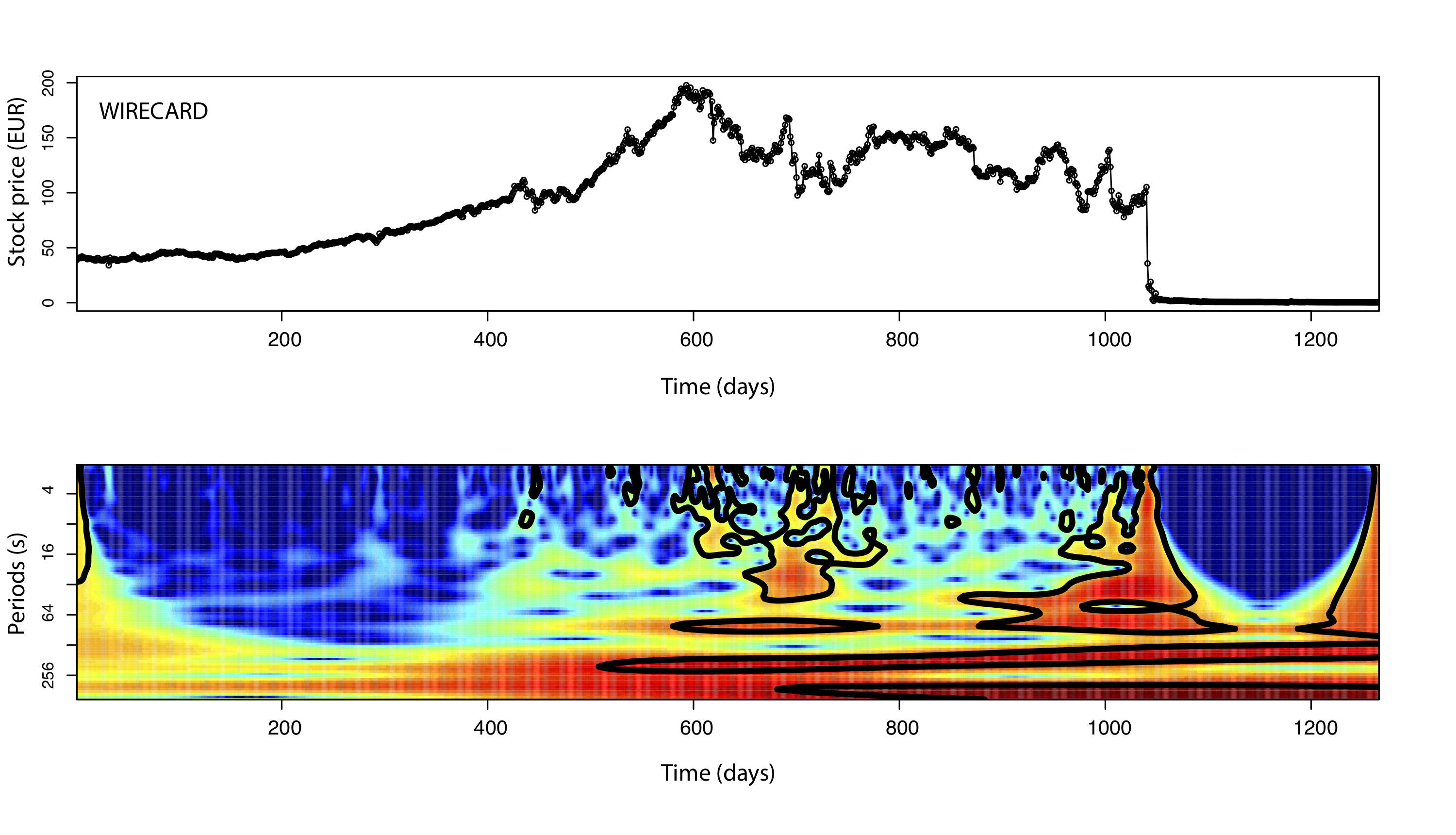

Supplement: S5 Fig — (TIFF) [file pone.0274165.s005.tiff]

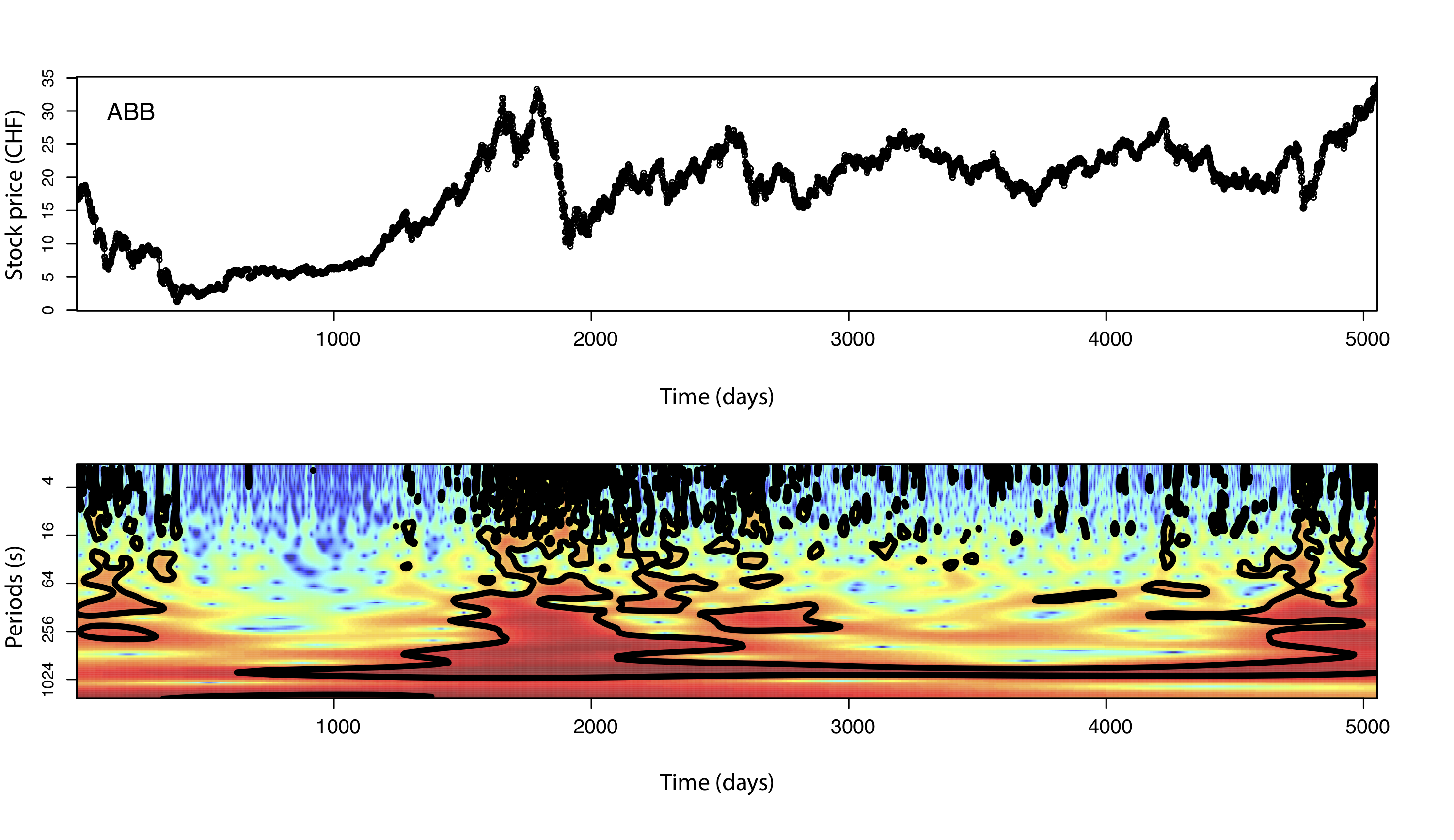

Supplement: S6 Fig — (TIFF) [file pone.0274165.s006.tiff]

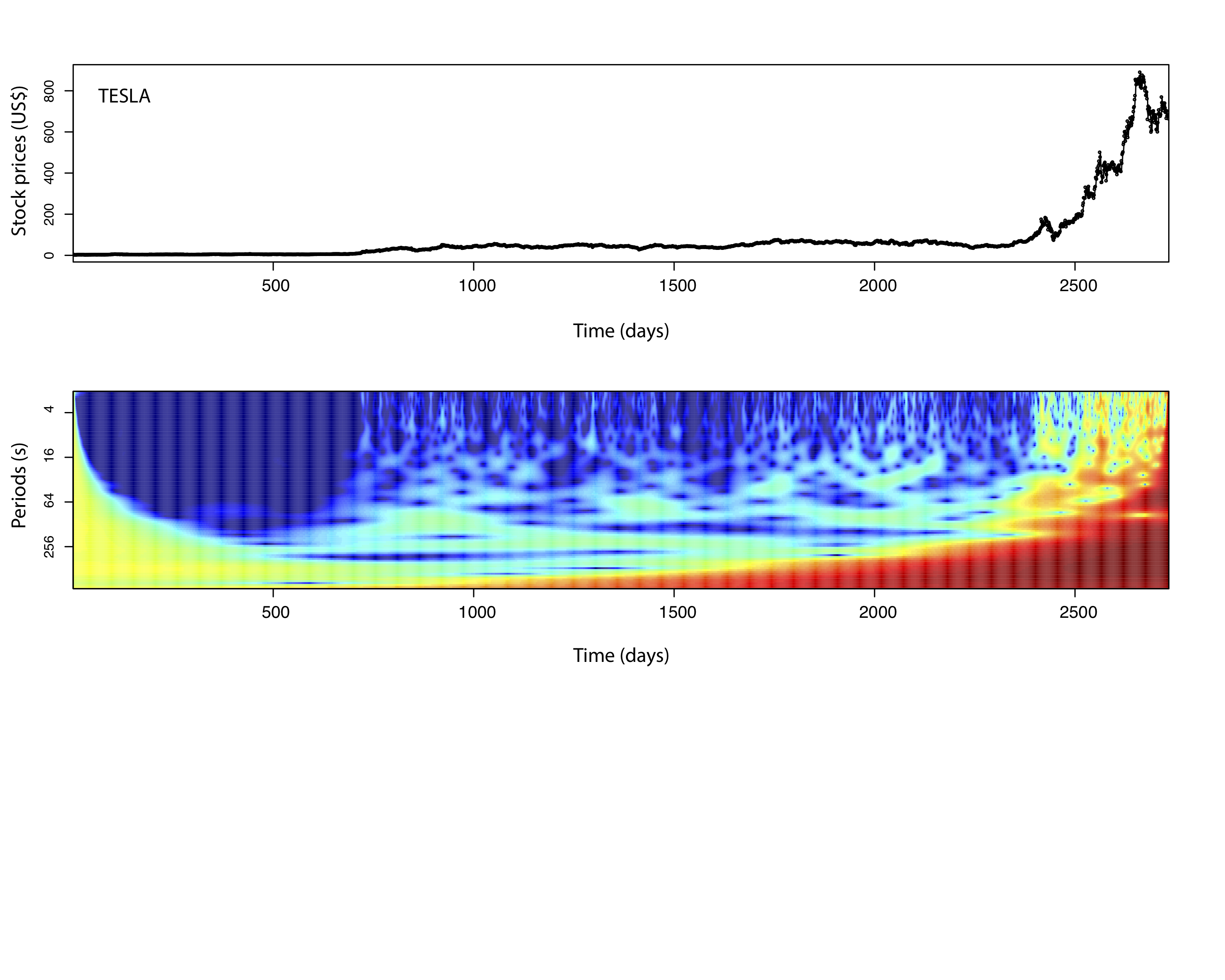

Supplement: S7 Fig — (TIFF) [file pone.0274165.s007.tiff]

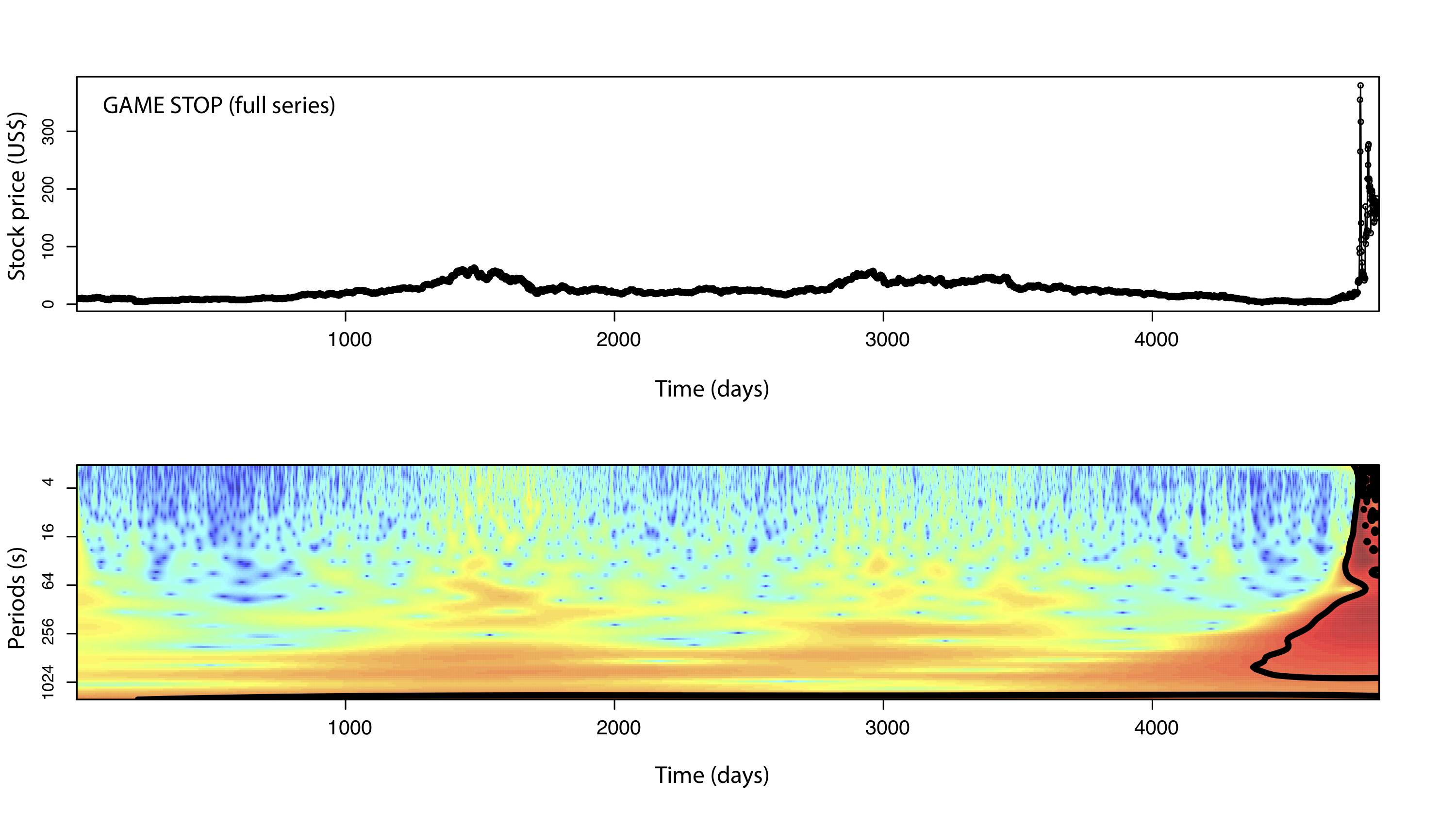

Supplement: S8 Fig — (TIFF) [file pone.0274165.s008.tiff]

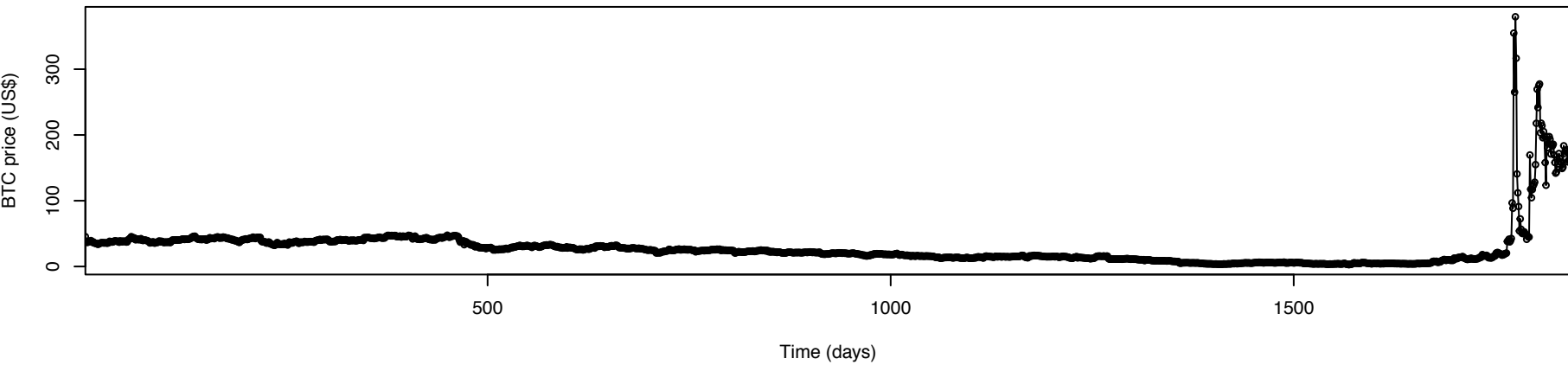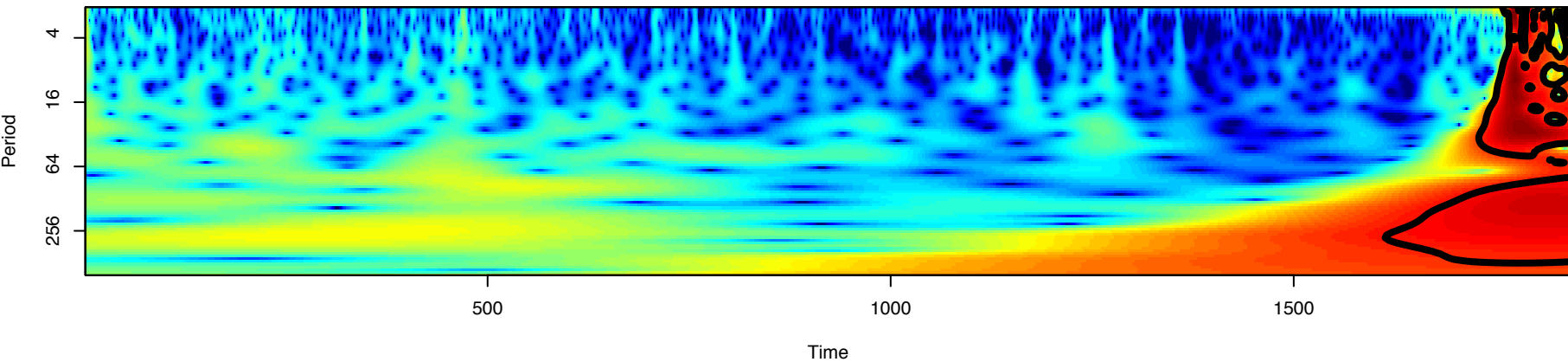

Supplement: S9 Fig — (PDF) [file pone.0274165.s009.pdf]
