# Supplementary material for: Bitcoin: A life in crises
Source: PLoS One. 2022 Sep 30;17(9):e0274165. doi: 10.1371/journal.pone.0274165 (PMC9524670; doi:10.1371/journal.pone.0274165)
Supplement: Attachment — Submitted filename: P1_Ans2Rev.docx [file pone.0274165.s014.docx]

Begin forwarded message:

**From:** "PLOS ONE" <[em@editorialmanager.com](mailto:em@editorialmanager.com)>
**Date:** 9 September 2019 at 10:09:37 CEST
**To:** "Nicolas Houlie" <[nhoulie@alumni.ethz.ch](mailto:nhoulie@alumni.ethz.ch)>
**Subject:** **PLOS ONE Decision: PONE-D-19-22666 - [EMID:1452ef76883c9dac]**
**Reply-To:** "PLOS ONE" <[plosone@plos.org](mailto:plosone@plos.org)>

[EXSCINDED]

PONE-D-19-22666
Bitcoin: a life in crises
PLOS ONE

Dear Dr. Houlie,

Thank you for submitting your manuscript to PLOS ONE. After careful consideration, we have decided that your manuscript does not meet our criteria for publication and must therefore be rejected.

Specifically:

Although you have an interesting and valuable paper and reviewers provided many valuable and constructive comments, the paper needs to be substantially improved before it can be considered for PLOS ONE. Since both Reviewer recommendations are Reject, I hope you can consider improving the manuscript accordingly and resubmitting it as a new article. And I would like to be the Academic Editor of the revised version again.

I am sorry that we cannot be more positive on this occasion, but hope that you appreciate the reasons for this decision.

Yours sincerely,

Baogui Xin, Ph.D.
Academic Editor
PLOS ONE


[Note: HTML markup is below. Please do not edit.]

Reviewers' comments:

Reviewer's Responses to Questions

**Comments to the Author**

1. Is the manuscript technically sound, and do the data support the conclusions?

The manuscript must describe a technically sound piece of scientific research with data that supports the conclusions. Experiments must have been conducted rigorously, with appropriate controls, replication, and sample sizes. The conclusions must be drawn appropriately based on the data presented.

Reviewer #1: No

Reviewer #2: No

2. Has the statistical analysis been performed appropriately and rigorously?

Reviewer #1: Yes

Reviewer #2: No

3. Have the authors made all data underlying the findings in their manuscript fully available?

The [PLOS Data policy](http://www.plosone.org/static/policies.action#sharing) requires authors to make all data underlying the findings described in their manuscript fully available without restriction, with rare exception (please refer to the Data Availability Statement in the manuscript PDF file). The data should be provided as part of the manuscript or its supporting information, or deposited to a public repository. For example, in addition to summary statistics, the data points behind means, medians and variance measures should be available. If there are restrictions on publicly sharing data—e.g. participant privacy or use of data from a third party—those must be specified.

Reviewer #1: No

Reviewer #2: No

The data are publicly available at the Yahoo website.

4. Is the manuscript presented in an intelligible fashion and written in standard English?

PLOS ONE does not copyedit accepted manuscripts, so the language in submitted articles must be clear, correct, and unambiguous. Any typographical or grammatical errors should be corrected at revision, so please note any specific errors here.

Reviewer #1: No

Reviewer #2: Yes

5. Review Comments to the Author

Please use the space provided to explain your answers to the questions above. You may also include additional comments for the author, including concerns about dual publication, research ethics, or publication ethics. (Please upload your review as an attachment if it exceeds 20,000 characters)

Reviewer #1: The paper finds that hockey-stick shaped pricing episodes occurred at least 8 times between 2012 and January 2018. The paper is interesting however I think that the current level of the paper is not suitable for the journal as it suffers from many minor and major issues.

- I couldn’t understand the “Bitcoin (BTC) price history:” at the beginning of the first highlights. I believe it is not needed. Likewise, here at the beginning of the abstract is not needed.

A1: Agreed I have modified these sentences.

- Abstract is not clear. What is the aim of the paper? What is the data period? What are the methodologies? None of them are given.

A2: Agreed. I have modified the abstract. I have added a sentence at the end of the abstract.

- The paper needs to benefit from a professional proofreading service. It is difficult to follow some sentences.

A3: I have asked a English native speaker to proof this manuscript

- The sentence, “As of today the combined Marketcap corresponding to the 5 largest CCs is > $200 bn ($120bn for BTC only); to be compared with $45 bn in march 2017 for BTC (Katsiampa, 2017).” this is sentence is totally non-informative as we don’t know what it is today. It is very easy to find the current market cap and make this sentence.

A4: This sentence has been modified

- Introduction is really difficult to follow. The paper jump from one field to another without connection. After reading the introduction, I have no insights about the aim, methodology, finding, and contribution of the paper.

A5: We have massively rewritten the introduction as the rest of the manuscript. We hope this version will be more comprehensible to the reviewers.

- The authors use both “I” and “we” in the text.

A6: all occurrences have been corrected. We are now two authors, so we are back to the “we” status.

- Data section: It is interesting that there is no time period information for the data.

A7: I have added these information in the data section and also in Table 1. We tried to be more consistent within the MS.

- Why the authors needed to mention the arbitrage in the data?

A8: I mentioned arbitrage data as the prices across platforms are different.

- Where does Eq1 come from? What are the notations inside?

A9: Corrected. I have added about the origin of this equation in the new version of the text.

- The citations are from CO2 concentration paper? Is this a method used in finance literature? And why or why not? “The CO2 concentration in the Earth atmosphere is well fitted by a yearly sampling rate”. By referring to that information, can we use it for Bitcoin? The connection is not clear.

A10: Yes each citation corresponds the previous part of the sentence. Fibonaci law and exponential price increases are sometimes used to detect speculative episodes; studies rather focusing on the stock analysis for private investors. Reference were added line 138. That is the objective of this article to show that we can use this function to describe BTC price too.

- It is also not clear that how we reach to 3c. There is no information about spillover till this point in the text.

A11: the paragraph 3c shows that it is not only one stock that is priced up but a whole class of assets. I have added some sentence to connect this better to the preceding and following sections. The paragraph about the other crypto currencies only shows tha confidence may be the principal driver of this BTC mania. Whether people are rational is not the problem anymore, we just need to find in which category of assets to classify it.

- There is no conclusion in the paper.

A12: I have modified the conclusion and rewritten the discussion completely.

Reviewer #2: The authors find some phenomenon and get some conclude in crypto-currencies, but I think the contribution is small and not novelty.

Summary: In this paper, the authors find that hockey-stick shaped pricing episodes occurred at least 8 times between 2012 and January 2018. The price in each crisis episodes is always following by a good value resistance. And the authors conclude that BTC may be classified as an illiquid asset.

Main concerns
(1) The authors don’t make specific efforts in understanding the asset classifying. They only find that the price in each crisis episodes is always following by a good value resistance.

A13: Nobody knows what is this instrument is backed by. One think it is backed up by strong confidence of players knowing about the real underlsing value. Anything can be considered from money laundering to human trafficking. As these activities are not knowing much crises these days, it might explain the resistance of bitcoin. For now we should focus on the level of risk carried out by it based on historical data. As the governance is opaque even though somewhat decentralized, observations may help define new regulations.

(2) The authors find that the curvey of BTC is similar to XAU and TRY, where quick change of price followed by a stagnation and then a sudden price readjustment after more information became available to a wider audience. But it is easy to find the similar phenomenon in many asset during a specific time period. The evidence is not sufficient to conclude that BTC is illiquidity.

A14: From a bank perspective and assuming market conditions are perfect, liquidity should be synonym of no-price change. We are far from that case here. The question about BTC is more related to which illiquidity: strong demand or strong offer. In this paper we focus on the first case. None of the examples showed in Figure 3 show the same shape as of BTC. The closest is XAU gold time-series but the amplitude of changes is much smaller.

I made the text less ambiguous.


Minor concerns:
1. There are some formatting issues. For example:
(I) I can not find the section 3.b in the article, but find the section "3.d Repetitive pattern through time" between section 3.a and 3.c.

A15: Corrected.

(II) There are too space in the last sentence in the section 3.a "The CO2 concentration in the Earth atmosphere is well fitted by a yearly sampling rate."

A16: Fixed

(III) We can not find any legend in Figure 2.

A17: Fixed. This is strange.

(IV) I can not find Figure 1.b.

A18: Figure 1 has two panels A and B. This sentence has been modified

Please check the correctness of the format.

A19: Done

2. The authors use a long paragraph to explain that the price curve look like a Hockey Stick Function in section 3.a and section 3.d. But what does it mean? It seems irrelevant with the topic of the article.

A20: We have added some explanation about the function itself near line 136.The origin of this curve is not clear, although it is commonly in the nature under various forms. For instance, similar curves are observed for the displacement of lanslides. Regarding BTC, one can speculate that the heavy redistribution of wealth into one asset may cause the BTC price evolution. Some could find some analogy with CO2 concentration causing climate change inducing the release of CO2 from reservoirs located previously unavailable (forest, permafrost, etc.). I think we deal here with some herd effects, many investors follow a first group which itself reinvest regularly.

6. PLOS authors have the option to publish the peer review history of their article ([what does this mean?](https://journals.plos.org/plosone/s/editorial-and-peer-review-process#loc-peer-review-history)). If published, this will include your full peer review and any attached files.


If you choose “no”, your identity will remain anonymous but your review may still be made public.


**Do you want your identity to be public for this peer review?** For information about this choice, including consent withdrawal, please see our [Privacy Policy](https://www.plos.org/privacy-policy).

Reviewer #1: No

Reviewer #2: No


[NOTE: If reviewer comments were submitted as an attachment file, they will be attached to this email and accessible via the submission site. Please log into your account, locate the manuscript record, and check for the action link "View Attachments". If this link does not appear, there are no attachment files to be viewed.]

- - - - -
For journal use only: PONEDEC3

*In compliance with data protection regulations, you may request that we remove your personal registration details at any time.* [*(Remove my information/details)*](https://www.editorialmanager.com/pone/login.asp?a=r)*. Please contact the publication office if you have any questions.*
