# Supplementary material for: Bitcoin: A life in crises
Source: PLoS One. 2022 Sep 30;17(9):e0274165. doi: 10.1371/journal.pone.0274165 (PMC9524670; doi:10.1371/journal.pone.0274165)
Supplement: Attachment — Submitted filename: comments.docx [file pone.0274165.s015.docx]

This is an interesting research paper regarding the Bitcoin market. After reviewing the whole manuscript, I think the paper needs to do some necessary revisions and resubmit to the Journal before the official publishing.

1. I have not seen any empirical results in the section of Introduction; thus, I strongly suggest the authors to add this part to Introduction. Besides, this section (Introduction) has not well illustrated the novelty of this paper.

2. I do suggest the authors to redo the figures in this paper. For example, the Horizontal axis in Figure 1 is number, shouldn't the horizontal axis be time?

3. I think the section 4.1 is quite confusing. The illustration is not convincing enough.

4. The structure of this paper is strange. Shouldn’t the conclusion be a last section of one paper?

5. The data in this paper is out of date. I do not think it is appropriate for current research. For example, the price of Bitcoin decreased sharply after April this year, it is of importance to discuss this time period rather than the data goes back a few years.
