# Supplementary material for: Bitcoin: A life in crises
Source: PLoS One. 2022 Sep 30;17(9):e0274165. doi: 10.1371/journal.pone.0274165 (PMC9524670; doi:10.1371/journal.pone.0274165)
Supplement: Attachment — Submitted filename: ANS_2_rev_R2.docx [file pone.0274165.s016.docx]

Begin forwarded message:

**From:** "PLOS ONE" <[em@editorialmanager.com](mailto:em@editorialmanager.com)>

**Subject: PLOS ONE Decision: Revision required [PONE-D-21-18020R1] - [EMID:21cc1c44b762cf87]**

**Date:** December 9, 2021 at 22:42:52 GMT+1

**To:** "Nicolas Houlie" <[nhoulie@alumni.ethz.ch](mailto:nhoulie@alumni.ethz.ch)>

**Reply-To:** "PLOS ONE" <[plosone@plos.org](mailto:plosone@plos.org)>

PONE-D-21-18020R1

BITCOIN: a life in crises

PLOS ONE

Dear Dr. Houlie,

Thank you for submitting your manuscript to PLOS ONE. After careful consideration, we feel that it has merit but does not fully meet PLOS ONE’s publication criteria as it currently stands. Therefore, we invite you to submit a revised version of the manuscript that addresses the points raised during the review process.

Though Reviewer 2 rejected PONE-D-21-26729, the reviewer provided many valuable and constructive comments. Considering three reviewers’ useful comments and the interesting topic of the manuscript, I would like to give you a chance to revise your manuscript during the special period. The revised manuscript will undergo the next round of review by the same reviewers.

Please submit your revised **manuscript by Jan 23 2022 11:59PM. If you will need** more time than this to complete your revisions, please reply to this message or contact the journal office at [plosone@plos.org](mailto:plosone@plos.org). When you're ready to submit your revision, log on to <https://www.editorialmanager.com/pone/> and select the 'Submissions Needing Revision' folder to locate your manuscript file.

Please include the following items when submitting your revised manuscript:

- A rebuttal letter that responds to each point raised by the academic editor and reviewer(s). You should upload this letter as a separate file labeled 'Response to Reviewers'.
- A marked-up copy of your manuscript that highlights changes made to the original version. You should upload this as a separate file labeled 'Revised Manuscript with Track Changes'.
- An unmarked version of your revised paper without tracked changes. You should upload this as a separate file labeled 'Manuscript'.

If you would like to make changes to your financial disclosure, please include your updated statement in your cover letter. Guidelines for resubmitting your figure files are available below the reviewer comments at the end of this letter.

If applicable, we recommend that you deposit your laboratory protocols in [protocols.io](http://protocols.io) to enhance the reproducibility of your results. [Protocols.io](http://Protocols.io) assigns your protocol its own identifier (DOI) so that it can be cited independently in the future. For instructions see: [https://journals.plos.org/plosone/s/submission-guidelines#loc-laboratory-protocols](https://journals.plos.org/plosone/s/submission-guidelines#loc-laboratory-protocols). Additionally, PLOS ONE offers an option for publishing peer-reviewed Lab Protocol articles, which describe protocols hosted on [protocols.io](http://protocols.io). Read more information on sharing protocols at <https://plos.org/protocols?utm_medium=editorial-email&utm_source=authorletters&utm_campaign=protocols>.

We look forward to receiving your revised manuscript.

Kind regards,

Baogui Xin, Ph.D.
Academic Editor
PLOS ONE

[Note: HTML markup is below. Please do not edit.]

Reviewers' comments:

Reviewer's Responses to Questions

**Comments to the Author**

1. If the authors have adequately addressed your comments raised in a previous round of review and you feel that this manuscript is now acceptable for publication, you may indicate that here to bypass the “Comments to the Author” section, enter your conflict of interest statement in the “Confidential to Editor” section, and submit your "Accept" recommendation.

Reviewer #1: (No Response)

Reviewer #2: All comments have been addressed

2. Is the manuscript technically sound, and do the data support the conclusions?

The manuscript must describe a technically sound piece of scientific research with data that supports the conclusions. Experiments must have been conducted rigorously, with appropriate controls, replication, and sample sizes. The conclusions must be drawn appropriately based on the data presented.

Reviewer #1: Yes

Reviewer #2: No

3. Has the statistical analysis been performed appropriately and rigorously?

Reviewer #1: Yes

Reviewer #2: No

We disagree. See some examples below:

- We have computed robustness test such as RMS values and they are shown in Table 1. This table is included in the current version of the manuscript and in ALL its previous version.
- For the Bendford analysis near line 120 (to 123), we carried Chi2 tests. *“Chi^2^ test analysis shows that the prices of BTC do not comply with Benford’s law (χ^2^=357 for n=8) as the occurrence of “6”s for the first digit is occurring too many times (approx. +50%; Figure 3).”*
- Stats on price correlations, near line 126: *“At last, we chose Ethereum as a reference because of its different governance mechanism and because of the high correlation between ETH and BTC prices (r^2^=0.91, N=180) over the last six months.”*

4. Have the authors made all data underlying the findings in their manuscript fully available?

The [PLOS Data policy](http://www.plosone.org/static/policies.action#sharing) requires authors to make all data underlying the findings described in their manuscript fully available without restriction, with rare exception (please refer to the Data Availability Statement in the manuscript PDF file). The data should be provided as part of the manuscript or its supporting information, or deposited to a public repository. For example, in addition to summary statistics, the data points behind means, medians and variance measures should be available. If there are restrictions on publicly sharing data—e.g. participant privacy or use of data from a third party—those must be specified.

Reviewer #1: Yes

Reviewer #2: Yes

5. Is the manuscript presented in an intelligible fashion and written in standard English?

PLOS ONE does not copyedit accepted manuscripts, so the language in submitted articles must be clear, correct, and unambiguous. Any typographical or grammatical errors should be corrected at revision, so please note any specific errors here.

Reviewer #1: Yes

Reviewer #2: Yes

6. Review Comments to the Author

Please use the space provided to explain your answers to the questions above. You may also include additional comments for the author, including concerns about dual publication, research ethics, or publication ethics. (Please upload your review as an attachment if it exceeds 20,000 characters)

Reviewer #1: This is an interesting research paper regarding the Bitcoin market. After reviewing the whole manuscript, I think the paper needs to do some necessary revisions before the official publishing.

1. Discussion should be the part to illustrate the rationality of the results. However, in the current manuscript, the current discussion seems to the outlook for the future. I suggest the authors to revise the current discussion.

We have rewritten the conclusion and the discussion by merging them and we did regorganize the text. This new version highlights more clearly findings, limitations and perspectives.

2. Descriptions should make some revisions. For example, “Finally” in Line 277 and “Finally” in Line 280.

We have browsed through the entire article to polish it one more time. See document with track changes.

Reviewer #2: This article investigated the Bitcoin price dynamics and found that there were eight events with similar durations during the sample period. Studying the price of Bitcoin is a very interesting topic, but I think the authors’ research is not innovative enough and contributions are not enough, and the research design is not rigorous enough.

Main concerns
1. The authors should emphasize the research motivation.

We refer there to the line number of the document assembled by Plos one PDF engine named “PONE-D-21-18020-R1.pdf”:

Line 40: “*classifying BTC (and some of the other CCs) for risk assessment remains necessary”*

Research motivation are indicated between lines 75 and 77

Why are you writing this article?

We refer there to the line number of the document assembled by Plos one PDF engine named “PONE-D-21-18020-R1.pdf”.

Why are we writing this article: lines 60 onwards. *“This has left banks and other financial services firms scrambling to deploy financial services (custodianship, cold storage, BTC payment services) and investment products (like funds, derivatives, and complex structured notes) into the market to capitalize on this trend, which in turn exposed them to non-trivial challenges, both regulatory and economical.”*

What conclusion do you want to get?

We refer there to the line number of the document assembled by Plos one PDF engine named “PONE-D-21-18020-R1.pdf”.

What conclusions we want to get: lines 75 to 77

2. The conclusion of the article is not credible. The author said that a function similar to the Fibonacci sequence can be used to approximate the price of Bitcoin. I find it hard to believe, and the author did not make further analysis.

First, everywhere in the manuscript we point to Fibonacci sequence as “similar to”, “parented to”. We use this analogy to help the reader understanding the nature/shape of the function.

Second, we do not need to “believe” anything: we have fitted synthetic functions to observed data. Also in scientific domain, we need to convince you with numbers and evidences. As it seems the R2 did not read this version of the manuscript, it make our task more difficult.

Third. we show the fits (consistency between price observed and the model) in Figures 5.x and show the RMS values in the Table 1. We have gathered 12+ observations to support our hypothesis.

3. The research design of the article is not rigorous. The authors’ conclusions all come from observations and intuitions of price dynamics, and they have not analyzed and discussed the conclusions in an economic sense.

See comment above and below. We now highlight in the new discussion what would be needed to understand better the cause (if unique) of a BTC price surge.

In addition, the author did not do a robustness test. Will the conclusion of the paper still hold for the period after January 1, 2020?

Shall we “believe” R2 read the latest version of this manuscript for real? This comment refers to draft PONE-S-21-22152 (end of dataset at 01 Jan 2020). It seems that Reviewer R2 did not read the latest submission (we have added data/results for the period post Jan 2020 in the last submission). Isn’t this a problem that R2 did not read the manuscript ?

4. The writing of the paper is also not standardized. The table in the text is not a standard three-line table,

We have modified the table layout to match this style

the letters in the formula are not explained further, and

We explain the letters in Eq. 2 on lines 174 and lines 178. We refer here to line numbers of MS PONE-D-21-18020_R2

the abscissa of the time series graph is not indicated by date. Therefore, I think this is not a qualified academic paper.

Figure 1 has date abscissa, as doFigure 5a – 5 d and also Figure 6. All mentioned figures are time-series. Those Figures are the only figures where dates are relevant to be seen. Maybe R2 was reading a previous version of the manuscript.

7. PLOS authors have the option to publish the peer review history of their article ([what does this mean?](https://journals.plos.org/plosone/s/editorial-and-peer-review-process#loc-peer-review-history)). If published, this will include your full peer review and any attached files.


If you choose “no”, your identity will remain anonymous but your review may still be made public.


**Do you want your identity to be public for this peer review?** For information about this choice, including consent withdrawal, please see our [Privacy Policy](https://www.plos.org/privacy-policy).

Reviewer #1: No

Reviewer #2: No

[NOTE: If reviewer comments were submitted as an attachment file, they will be attached to this email and accessible via the submission site. Please log into your account, locate the manuscript record, and check for the action link "View Attachments". If this link does not appear, there are no attachment files.]

While revising your submission, please upload your figure files to the Preflight Analysis and Conversion Engine (PACE) digital diagnostic tool, <https://pacev2.apexcovantage.com/>. PACE helps ensure that figures meet PLOS requirements. To use PACE, you must first register as a user. Registration is free. Then, login and navigate to the UPLOAD tab, where you will find detailed instructions on how to use the tool. If you encounter any issues or have any questions when using PACE, please email PLOS at [figures@plos.org](mailto:figures@plos.org). Please note that Supporting Information files do not need this step.

*In compliance with data protection regulations, you may request that we remove your personal registration details at any time.* [*(Remove my information/details)*](https://www.editorialmanager.com/pone/login.asp?a=r)*. Please contact the publication office if you have any questions.*
