# Supplementary material for: Bitcoin: A life in crises
Source: PLoS One. 2022 Sep 30;17(9):e0274165. doi: 10.1371/journal.pone.0274165 (PMC9524670; doi:10.1371/journal.pone.0274165)
Supplement: Attachment — Submitted filename: ANS_2_comments_R3.docx [file pone.0274165.s017.docx]

This is an interesting research paper regarding the Bitcoin market. After reviewing the whole manuscript, I think the paper needs to do some necessary revisions and resubmit to the Journal before the official publishing.

1. I have not seen any empirical results in the section of Introduction; thus, I strongly suggest the authors to add this part to Introduction. Besides, this section (Introduction) has not well illustrated the novelty of this paper.

**ANSWER 1: We like to see Results in the results section. This introduction has been modified in that sense already.**

2. I do suggest the authors to redo the figures in this paper. For example, the Horizontal axis in Figure 1 is number, shouldn't the horizontal axis be time?

**ANSWER 2: This comment has been addressed in the revision R1. We show our answer made at the time below. The Figure 1 HAS time labels since 2 versions (from this summer). This figure has not been modified in version R2. See screenshot below (from the answer to reviewer R1 version)**

3. I think the section 4.1 is quite confusing. The illustration is not convincing enough.

**ANSWER 3: This comment has been addressed in the revision R1. We show our answer made at the time below. This section has been rewritten at the time. See screenshot below (from the answer to reviewer R1 version)**

4. The structure of this paper is strange. Shouldn’t the conclusion be a last section of one paper?

**ASNWER 4: The is no conclusion section in our paper anymore. R2 does not read the correct version of the manuscript.**

5. The data in this paper is out of date. I do not think it is appropriate for current research. For example, the price of Bitcoin decreased sharply after April this year, it is of importance to discuss this time period rather than the data goes back a few years.

**ASNWER 5: R2 comment is wrong, but it is similar to his previous comment about the extent of our study stopping in Jan 2020 when our dataset ended with the peak of Jan 2021 (last peak at the time of submission in June 2021).**

**Since April there was more price peaks (September/October 2021). Of course we could continue adding more peaks to our study. It does make it even more robust but we would never stop adding them….**

**Nevertheless, we added a sentence about confidence reversal in the new text. In the current manuscript, we already discuss at length about the confidence of the investors (in the current version of the paper…). If R2 reads the latest version of the paper, he may be convinced.**
