# Supplementary material for: Bitcoin: A life in crises
Source: PLoS One. 2022 Sep 30;17(9):e0274165. doi: 10.1371/journal.pone.0274165 (PMC9524670; doi:10.1371/journal.pone.0274165)
Supplement: Attachment — Submitted filename: 20220614_ANS_2_rev.docx [file pone.0274165.s018.docx]

Begin forwarded message:

**From:** "PLOS ONE" <[em@editorialmanager.com](mailto:em@editorialmanager.com)>

**Subject: Editor Decision - Major Revision - [EMID:f931415480bdf69d]**

**Date:** 9 June 2022 at 02:06:48 CEST

**To:** "Nicolas Houlie" <[nhoulie@alumni.ethz.ch](mailto:nhoulie@alumni.ethz.ch)>

**Reply-To:** "PLOS ONE" <[plosone@plos.org](mailto:plosone@plos.org)>

PONE-D-21-18020R3
BITCOIN: a life in crises
PLOS ONE

Dear Dr. Houlie,

Thank you for submitting your manuscript to PLOS ONE. After careful consideration, we feel that it has merit but does not fully meet PLOS ONE’s publication criteria as it currently stands. Therefore, we invite you to submit a revised version of the manuscript that addresses the points raised during the review process.

Though Reviewer 5 rejected PONE-D-21-18020R3, the reviewer provided some valuable and constructive comments. Considering two reviewers' useful comments and the interesting topic of the manuscript, I would like to give you a chance to revise your manuscript during the special period. The revised manuscript will undergo the next round of review by the same reviewers.

Please submit your revised manuscript by Jul 17 2022 11:59PM. If you will need more time than this to complete your revisions, please reply to this message or contact the journal office at [plosone@plos.org](mailto:plosone@plos.org). When you're ready to submit your revision, log on to <https://www.editorialmanager.com/pone/> and select the 'Submissions Needing Revision' folder to locate your manuscript file.

Please include the following items when submitting your revised manuscript:
A rebuttal letter that responds to each point raised by the academic editor and reviewer(s). You should upload this letter as a separate file labeled 'Response to Reviewers'.

A marked-up copy of your manuscript that highlights changes made to the original version. You should upload this as a separate file labeled 'Revised Manuscript with Track Changes'.

An unmarked version of your revised paper without tracked changes. You should upload this as a separate file labeled 'Manuscript'.

If you would like to make changes to your financial disclosure, please include your updated statement in your cover letter. Guidelines for resubmitting your figure files are available below the reviewer comments at the end of this letter.

If applicable, we recommend that you deposit your laboratory protocols in [protocols.io](http://protocols.io) to enhance the reproducibility of your results. [Protocols.io](http://Protocols.io) assigns your protocol its own identifier (DOI) so that it can be cited independently in the future. For instructions see: <https://journals.plos.org/plosone/s/submission-guidelines#loc-laboratory-protocols>. Additionally, PLOS ONE offers an option for publishing peer-reviewed Lab Protocol articles, which describe protocols hosted on [protocols.io](http://protocols.io). Read more information on sharing protocols at <https://plos.org/protocols?utm_medium=editorial-email&utm_source=authorletters&utm_campaign=protocols>.

We look forward to receiving your revised manuscript.

Kind regards,

Baogui Xin, Ph.D.
Academic Editor
PLOS ONE

[Note: HTML markup is below. Please do not edit.]

Reviewers' comments:

Reviewer's Responses to Questions

Comments to the Author

1. If the authors have adequately addressed your comments raised in a previous round of review and you feel that this manuscript is now acceptable for publication, you may indicate that here to bypass the “Comments to the Author” section, enter your conflict of interest statement in the “Confidential to Editor” section, and submit your "Accept" recommendation.

**Reviewer #4: All comments have been addressed

Reviewer #5: All comments have been addressed**

**ANSWER 01: From this comment we consider we do not need to answer the comments attached to the current revision request. It seems to us that this file is inherited from another revision set and these comments have been answered in September 2021. We consider this as a mistake of the editor assistant. However, we update our answer to question 5 as highlighted in the cover letter.**

2. Is the manuscript technically sound, and do the data support the conclusions?

The manuscript must describe a technically sound piece of scientific research with data that supports the conclusions. Experiments must have been conducted rigorously, with appropriate controls, replication, and sample sizes. The conclusions must be drawn appropriately based on the data presented.

Reviewer #4: Yes

Reviewer #5: Partly

3. Has the statistical analysis been performed appropriately and rigorously?

Reviewer #4: Yes

Reviewer #5: No

4. Have the authors made all data underlying the findings in their manuscript fully available?

The PLOS Data policy requires authors to make all data underlying the findings described in their manuscript fully available without restriction, with rare exception (please refer to the Data Availability Statement in the manuscript PDF file). The data should be provided as part of the manuscript or its supporting information, or deposited to a public repository. For example, in addition to summary statistics, the data points behind means, medians and variance measures should be available. If there are restrictions on publicly sharing data—e.g. participant privacy or use of data from a third party—those must be specified.

Reviewer #4: Yes

Reviewer #5: Yes

5. Is the manuscript presented in an intelligible fashion and written in standard English?

PLOS ONE does not copyedit accepted manuscripts, so the language in submitted articles must be clear, correct, and unambiguous. Any typographical or grammatical errors should be corrected at revision, so please note any specific errors here.

Reviewer #4: Yes

Reviewer #5: Yes

6. Review Comments to the Author

Please use the space provided to explain your answers to the questions above. You may also include additional comments for the author, including concerns about dual publication, research ethics, or publication ethics. (Please upload your review as an attachment if it exceeds 20,000 characters)

**Reviewer #4: This is an interesting paper. It is well motivated and generally well written. The topic is interesting and the results can have interesting policy implications for both policymakers and investors. Compared with the previous two versions, I can see the quality of this paper improves a lot. I therefore recommend that the paper be published in PLOS ONE. Congratulations to the authors.**

**ANSWER 02: We thank R1 for his positive feedback. We continued to research in the domain of price reduction since the last version. We show this latest finding in the new version and mention this in the discussion of the paper.**

**Reviewer #5: It seems a very simple use of some equations. The topic at the beginning of the papar is very interesting but fail to be technically sound. I do not find a contribution around some diffeent ideas or matehmatically different. The conclusions are very naive.**

**ANSWER 03: We proposed in our paper that price increases and stabilization were driven at least partially by confidence. Recent events (price fall) have shown that confidence is driving the dynamics of the BTC price (wrt. Luna) and BTC price was not free being impacted by adverse market conditions (central banks interest rises). Since the last version of the manuscript, we have added a figure showing that our model is also true for price decreases (including the one of 15 Jun 2022). We thank R5 for his comments and hope the latest additional improvements will allow decrease the perceived level of naiveness of our research.**

7. PLOS authors have the option to publish the peer review history of their article (what does this mean?). If published, this will include your full peer review and any attached files.


If you choose “no”, your identity will remain anonymous but your review may still be made public.


Do you want your identity to be public for this peer review? For information about this choice, including consent withdrawal, please see our Privacy Policy.

Reviewer #4: No

Reviewer #5: No


[NOTE: If reviewer comments were submitted as an attachment file, they will be attached to this email and accessible via the submission site. Please log into your account, locate the manuscript record, and check for the action link "View Attachments". If this link does not appear, there are no attachment files.]

While revising your submission, please upload your figure files to the Preflight Analysis and Conversion Engine (PACE) digital diagnostic tool, <https://pacev2.apexcovantage.com/>. PACE helps ensure that figures meet PLOS requirements. To use PACE, you must first register as a user. Registration is free. Then, login and navigate to the UPLOAD tab, where you will find detailed instructions on how to use the tool. If you encounter any issues or have any questions when using PACE, please email PLOS at [figures@plos.org](mailto:figures@plos.org). Please note that Supporting Information files do not need this step.

__________________________________________________
In compliance with data protection regulations, you may request that we remove your personal registration details at any time.  (Use the following URL: <https://www.editorialmanager.com/pone/login.asp?a=r>). Please contact the publication office if you have any questions.

**We have copied the comment of R1 below and answered to each of them. Some of the these comments were already addressed (answer version as of Sep. 4, in blue). We updated one of our answer (question 5).**

This is an interesting research paper regarding the Bitcoin market. After reviewing the whole manuscript, I think the paper needs to do some necessary revisions and resubmit to the Journal before the official publishing.

1. I have not seen any empirical results in the section of Introduction; thus, I strongly suggest the authors to add this part to Introduction. Besides, this section (Introduction) has not well illustrated the novelty of this paper.

Since the last submission we have modified Figure 1 (price time-series). We have added another figure (new Figure 4) showing the self-similarity of the BTC price time-series. Rewriting the introduction allowed to make the data presentation and motivations more clear.

We think the data should be presented not before the data section. We also to keep rsults within the result section to make a difference between what is osbvious and what is new in the paper. The new figure would be seen as an empirical result.

Novelty: we have rewritten many sections of the article (see attached doc showing differences), we have tried to highlight general goals in many place. Goals are specified and reinforced near lines 73 -109 of the introduction.

2. I do suggest the authors to redo the figures in this paper. For example, the Horizontal axis in Figure 1 is number, shouldn't the horizontal axis be time?

We have redone some figures:

-Figure 1 is new and with both dates and numbers of days

-(old) Figure 4 (now Figure 5) has been expanded to be more clear. We can see better each data fit.

-Figure 4 (new, self-similarity) axes includes both dates and day number

3. I think the section 4.1 is quite confusing. The illustration is not convincing enough.

Agreed, we have modified the text: added a section on liquidity and also justified the choice of assets used for comparison in more detail. In short, this shows that BTC is nothing new under the sun. Please note that Figure 4 is now a good complement of the new figure 5 (Former Figure 4 showing the data fits) showing the self-similarity of the BTC price time-series.

4. The structure of this paper is strange. Shouldn’t the conclusion be a last section of one paper?

We would have liked to keep the discussion last to reopen the debate with the community (maybe this is an habit the physics community) but we obey to R1.

5. The data in this paper is out of date. I do not think it is appropriate for current research. For example, the price of Bitcoin decreased sharply after April this year, it is of importance to discuss this time period rather than the data goes back a few years.

We have updated the data set used to capture more recent events. 14 events are now presented (from the 9 in the previous version of the paper). Regarding April 2021, I think you have in mind the jokes and changes of mind of Elon Musk regarding Tesla payment capabilities. “Elon Musk effect” is not the same as the one we described. We would like to keep such an event on the side as we do not consider this kind of market behaviour is well accepted by regulating bodies in particular and does not allow learning on the nature of an asset in general. We have already mentioned the TSLA bitcoin adoption (and reversal).

~~We have tried to play with the data during sharp decreases but could not fit the function to it.~~

We could fit some prices decreases (6 of them, shown in Figure 8 of the new manuscript) which happened since this comment has been made. We have now documented price decreases for the first time with the same function (flipped). We also point in the manuscript that the period of price development is shorter than for price increases.
